# The audiovisual structure of onomatopoeias: An intrusion of real-world physics in lexical creation

**DOI:** 10.1371/journal.pone.0193466

**Published:** 2018-03-21

**Authors:** Alan Taitz, M. Florencia Assaneo, Natalia Elisei, Mónica Trípodi, Laurent Cohen, Jacobo D. Sitt, Marcos A. Trevisan

**Affiliations:** 1 Department of Physics, IFIBA-University of Buenos Aires, Buenos Aires, Argentina; 2 Department of Psychology, New York University, New York, United States of America; 3 Medicine School, University of Buenos Aires, Buenos Aires, Argentina; 4 Department of Linguistics, University of Buenos Aires, Buenos Aires, Argentina; 5 INSERM U1127, Institut du Cerveau et de la Moelle Épinière, Paris, France; 6 CNRS UMR 7225, Institut du Cerveau et de la Moelle Épinière, Paris, France; 7 Sorbonne Universités, UPMC Univ Paris 06, Paris, France; 8 AP-HP, Groupe Hospitalier Pitié-Salpêtrière, Departament of Neurology, Paris, France; Leiden University, NETHERLANDS

## Abstract

Sound-symbolic word classes are found in different cultures and languages worldwide. These words are continuously produced to code complex information about events. Here we explore the capacity of creative language to transport complex multisensory information in a controlled experiment, where our participants improvised onomatopoeias from noisy moving objects in audio, visual and audiovisual formats. We found that consonants communicate movement types (slide, hit or ring) mainly through the manner of articulation in the vocal tract. Vowels communicate shapes in visual stimuli (spiky or rounded) and sound frequencies in auditory stimuli through the configuration of the lips and tongue. A machine learning model was trained to classify movement types and used to validate generalizations of our results across formats. We implemented the classifier with a list of cross-linguistic onomatopoeias simple actions were correctly classified, while different aspects were selected to build onomatopoeias of complex actions. These results show how the different aspects of complex sensory information are coded and how they interact in the creation of novel onomatopoeias.

## Introduction

The arbitrary nature of the linguistic sign has been an idealized notion of modern linguistics that served to explore the unlimited expressive power of language [1]. As research evolved, the fully arbitrary nature of the link between form and meaning has been called into question, opening the scientific exploration of this relationship. The results of those investigations show that nonarbitrary associations are not limited to exceptional cases or to specific word classes: a striking demonstration of this comes from a statistical analysis performed over two-thirds of the world’s languages, revealing that unrelated languages use the same sounds for specific referents [2].

Iconicity is a prominent form of non-arbitrariness, in which different aspects of the form and the meaning of words are related by perceptuomotor analogies [3]. Onomatopoeias are privileged objects to study iconic properties of spoken words. Just like any other word, onomatopoeias are embedded in the language and have to adapt to the local phonology, assuming arbitrary properties. However, they also they tend to maximize the similarity between speech sounds and the sounds of the actions they represent, preserving parts of the onomatopoeic structure across languages [4].

Beyond sound imitation stand the mimetic words, a more general class used to express actions where sound is not essential [5]. It has been suggested that these words, found in Japanese, are related to the interaction between the body and the linguistic sound system: mimetic words use sounds to imitate sensations including body movements, touch, vision, smell, taste, and sound [6]. Interestingly, this kind of words were found in many other languages [7–9]. The concept of ideophone was then coined to characterize 'a word, often onomatopoeic, which describes a predicate, qualificative or adverb in respect to manner, color, sound, smell, action, state or intensity' [10]. For instance, operations like lengthening and reduplication tend to evoke repetition and multiplicity, while monosyllabic forms tend to evoke unitary events [11]. Ideophones rise from the rest of the words to depict sensory imagery; beyond the mere imitation of sounds, their structure map onto the aspectual and motion structure of events [12].

A vast corpus of literature supports not only that sound-symbolic word classes are found in different cultures and languages worldwide, but also that these words, in continuous production [11], are naturally created to code complex information about events. Is it possible to recreate the formation and transmission of complex sensory information through words? Here we explore this capacity of creative language to transport complex multisensory information in a controlled experiment.

Form-meaning iconicity has been extensively studied in controlled conditions. In one famous experiment, Köhler [13] reported a strong bias of novel words used to label rounded and spiky images, which is known as the bouba/kiki effect. The exploration of this effect allowed unveiling the nature of the bias in terms of vowels and consonants preferred for naming either shape type [14–16]. Another type of form-meaning correspondence relates the size of the objects with the spoken words used to label them. As in the case of shapes, big and small images are associated to novel words characterized by specific sets of vowels and consonants. In this case, there is also a physical link between the size of the objects and the types of sounds they produce: big objects typically produce sounds of lower frequencies than small ones. Therefore, the correspondence of the object size on words can also be linked to direct imitation of acoustical features [17,18]. Other acoustical features such as the pitch were also shown to convey information describing the direction of motion of an object [19].

In most of these experimental studies on form-meaning correspondences, perceptual aspects of the events were considered in isolation: participants produce novel words to name static shapes [13,14,16,20], a single object performing different motions [19] or different sounds [4]. However, onomatopoeias imitate different objects in noisy interactions, i.e. they result of the integration of visual and auditory cues on moving objects. In this work we explored the structure of novel onomatopoeias created to describe events in multisensory scenarios. We used a group of images of different shapes and sizes performing three basic noisy physical events involving solid objects, which are acoustically correlated to the principal classes of phonemes [21].

We hypothesized that 1. different sensory modalities present specific phonological rules, and 2. within each sensory modality, a competition is established to communicate the features of a stimulus (i.e. the shape of the object and its movement type) that can be extracted from the phonological properties of the onomatopoeias. To explore these hypotheses, we created and analyzed a database of novel onomatopoeias using audio-visual stimuli of different interacting objects.

## Results

Nineteen participants freely created onomatopoeias based on movies representing one or two moving objects engaged in noisy physical interactions. The stimuli made use of objects of two shapes (rounded or spiky) and two sizes (big or small) performing three basic movements (hit, slide or ring). The sounds of hits, slides and rings associated with these movements each existed in one high-pitch (HF) and one low-pitch (LF) version (see Methods and S1 File for videos).

**Fig 1:**
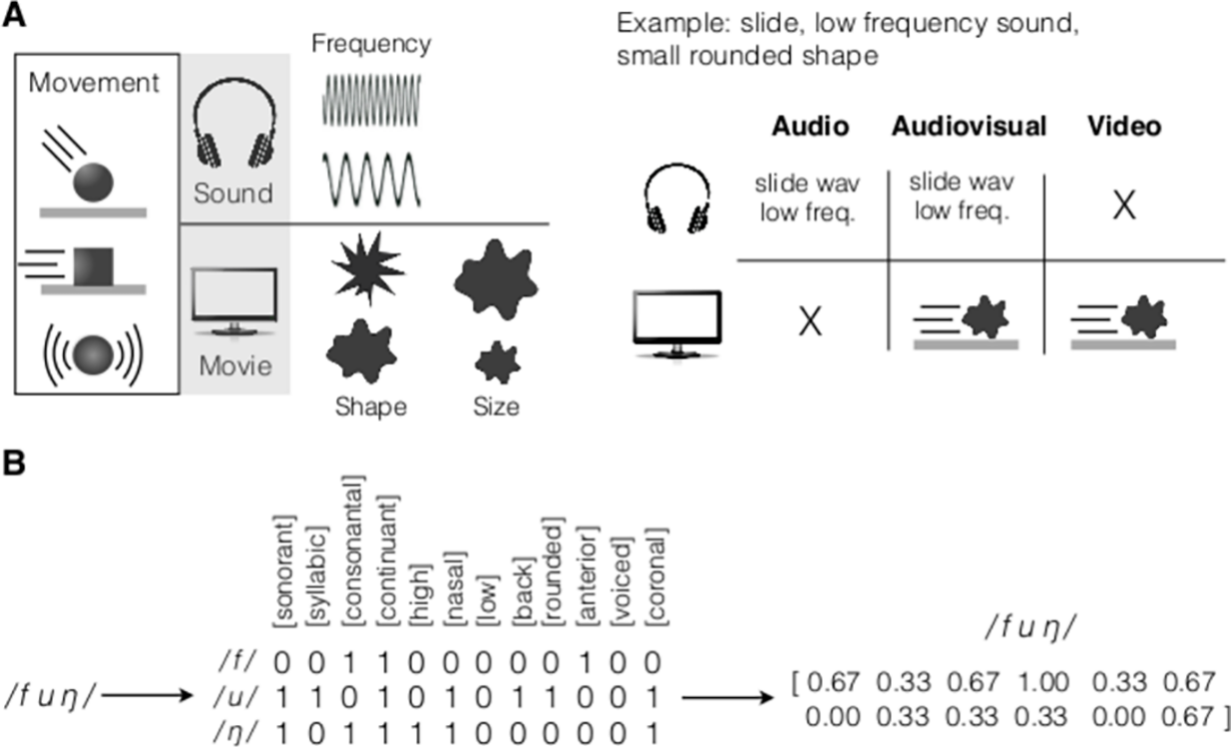
Participants created onomatopoeias from audio, visual and audiovisual stimuli of moving objects. Blocks of audio (A), visual (V) and audio-visual (AV) stimuli were constructed using objects of different shape (rounded or spiky) and size (big or small) performing movements (rings, hits or slides) with sounds of two different frequencies (high or low). **a.** The participants produced onomatopoeic sounds representing stimuli for all combinations of variables for each block. **b.** The onomatopoeias were transcribed to the International Phonetic Alphabet (IPA). The phonemes were associated with a binary 12-dimensional vector of low-level phonological features. **c.** Each onomatopoeia was characterized by the matrix of its phonemes in the phonological feature space, which was further averaged across phonemes to a final 12-dimensional vector.

Stimuli were presented to the participants in three sensory modalities: in the audiovisual (AV) format, every combination of shape, size and movement was presented with the two versions (HF and LF) of the corresponding sound. In the audio (A) and video (V) modalities, only the sounds and images were presented, respectively (Fig 1A). After each stimulus, the participants were asked to pronounce the onomatopoeic word that would better represent it, generating a database of recorded onomatopoeias.

### Phonological coding of onomatopoeias

We transcribed the recorded onomatopoeias into their constituent phonemes, using the symbols of the International Phonetic Alphabet [22]. For instance, the transcription of an onomatopoeia produced to describe an object sliding on a plane is the chain of phonemes fuŋ. Each phoneme was then broken down into its constituent *distinctive features*, which are the most basic phonological units in which phonemes can be decomposed [23]. The features used here were: *sonorant*, *syllabic*, *consonantal*, *continuant*, *nasal*, *high*, *low*, *back*, *round*, *anterior*, *coronal* and *voiced* (see Methods for details). Phonemes are uniquely specified by the presence or absence of these 12 features, which allowed us to represent each phoneme as a list of binary values 1 and 0. This operation thus maps every phoneme to a point in a 12-dimensional binary space (Fig 1B). In this representation, the first phoneme of the onomatopoeia fuŋ reads /f/ = (001100000100). This array says that the phoneme /f/ is produced when there is a difference between the air pressure inside and outside the vocal tract (not *sonorant*); it does not constitute a peak intensity of the syllable (not *syllabic*); the vocal tract is constricted (*consonantal*) with airflow passing through the oral (*continuant*) and not through the nasal (not *nasal*) tract; the body of the tongue is not raised towards the palate (not *high*), nor drawn down (not *low*) or retracted (not *back*); the lips are unrounded (not *rounded*); the constriction is located at the alveolar ridge (*anterior*) without raising the tongue blade toward the teeth (not *coronal*) and the vocal folds do not oscillate (not *voiced*).

In this representation, an onomatopoeia is associated with a matrix whose rows correspond to its phonemes and columns to their decomposition along the distinctive features. This is shown in Fig 1C for the case of the onomatopoeia fuŋ. Due to their variable number of phonemes, onomatopoeias are represented by matrices of a variable number of rows. To be able to compare them, we had to avoid dealing with mathematical objects of different dimensions, and averaged each matrix across phonemes. Each onomatopoeia was therefore mapped to a single 12-dimensional array whose values are continuous numbers between 0 and 1, as shown in the example of Fig 1C. This array represents the relative weights of the distinctive features used in the onomatopoeia.

We also characterized the onomatopoeias using the alternative system of *IPA features* [22]. In the IPA space, the complete corpus of phonemes is organized in two sets: vowels and consonants (S1 Fig).

Vowels are generated by different configurations of a non-constricted vocal tract. In the IPA space, vowels are classified in a 3-dimensional space according to the vertical (*heightness*) and horizontal (*backness*) positions of the tongue body, and the *roundness* of the lips. These features are not necessary binary. For instance, vowels have three possible levels of heightness: close, mid or open. The five dimensions of vowels considered here are: backness, heightness (close, mid and open) and roundness.

To generate a consonant, a constriction has to be imposed to the vocal tract. The acoustic properties of the consonant depend on the activation or inactivation of the vocal folds (*voiced*), the place of the constriction (from the larynx to the mouth: *radical*, *dorsal*, *coronal* or *labial*) and its manner of articulation: *plosives* (occluded vocal tract), *liquids* (partially occluded vocal tract), *fricatives* (constricted vocal tract) and *nasals* (open nasal tract). This makes a total of nine consonantal dimensions. Taking our previous example, in the IPA description, the phoneme /f/ is a labial fricative, non-voiced consonant.

The IPA features can be described in terms of distinctive features, as shown in dark and light gray respectively in S1 Fig: labials are represented by arrays of the form (xxxxxxxxx10x), fricatives by (0xx1xxxxxxxx), non-voiced by (xxxxxxxxxxx0) and consonants by (xx1xxxxxxxxxx).

IPA and distinctive features were included in our analyses in order to compare the efficiency of those systems in accounting for the iconic properties of onomatopoeias. Once each onomatopoeia thus translated in an array of features, we explored the links between stimulus properties and phonology.

### Sensitivity of phonological features to stimulus properties

In order to evaluate the sensitivity of phonological features to stimulus properties, we submitted the corpus of onomatopoeias to independent ANOVA analyses for each phonological feature, with subjects as random factor. In Table 1 we summarize the results for the different modalities: AV (movement, shape and sound as independent factors), A (movement and sound) and V (movement and shape). The p-values of each ANOVA were Bonferroni corrected for the 26 phonological dimensions (12 for the distinctive features and 14 for IPA features), to account for multiple comparisons (*p*<0.00038 = 0.01/26 was considered significant; bold face in Table 1).

**Table 1:** ANOVA feature analyses for audio, audiovisual and video sensory modalities.

				A				AV				V		
				Mov	Sound	Shape		Mov	Sound	Shape		Mov	Sound	Shape
DISTINCTIVE			**Sonant**	**<10**^**−4**^	0.1246			**<10**^**−4**^	0.4427	0.7027		0.0164		0.6257
			**Syllabic**	0.0024	0.0968			**<10**^**−4**^	0.0600	0.7481		**0.00032**		0.9580
			**Consonant**	**0.00013**	0.0254			**<10**^**−4**^	0.0025	0.8659		**<10**^**−4**^		0.6415
			**Continuant**	**<10**^**−4**^	0.1315			**<10**^**−4**^	0.2288	0.1175		**<10**^**−4**^		0.9355
			**Nasal**	**<10**^**−4**^	0.2397			**<10**^**−4**^	0.0013	0.3536		**<10**^**−4**^		0.9127
			**High**	0.7926	0.0342			**<10**^**−4**^	0.0065	0.6829		**<10**^**−4**^		0.0163
			**Low**	0.0043	**<10**^**−4**^			**<10**^**−4**^	**<10**^**−4**^	0.4490		0.1556		0.1345
			**Back**	0.0350	**<10**^**−4**^			**<10**^**−4**^	**<10**^**−4**^	0.9588		0.0092		0.5156
			**Round**	0.8169	**<10**^**−4**^			0.1304	**<10**^**−4**^	0.4913		0.0017		**<10**^**−4**^
			**Anterior**	0.9421	0.1616			**<10**^**−4**^	**<10**^**−4**^	0.8319		**<10**^**−4**^		0.0190
			**Coronal**	0.0609	**<10**^**−4**^			**<10**^**−4**^	**<10**^**−4**^	0.1915		0.0041		**<10**^**−4**^
			**Voiced**	**<10**^**−4**^	0.2409			**<10**^**−4**^	0.4835	0.4118		0.0166		0.0258
IPA	Cons	Place	**RADICAL**	0.1199	0.3546			**<10**^**−4**^	0.0136	0.4456		**<10**^**−4**^		0.2286
			**DORSAL**	0.0039	0.7696			**<10**^**−4**^	0.5994	0.2435		**<10**^**−4**^		0.0011
			**CORONAL**	**<10**^**−4**^	0.0101			**<10**^**−4**^	**<10**^**−4**^	0.4760		0.0711		0.0132
			**LABIAL**	**<10**^**−4**^	0.3538			**<10**^**−4**^	0.0230	0.3482		**<10**^**−4**^		**<10**^**−4**^
		Manner	**PLOSIVE**	**<10**^**−4**^	0.1315			**<10**^**−4**^	0.2288	0.1175		**<10**^**−4**^		0.9355
			**FRICATIVE**	**<10**^**−4**^	0.8912			**<10**^**−4**^	0.0555	0.3036		**<10**^**−4**^		0.5703
			**LIQUID**	**<10**^**−4**^	0.7171			**<10**^**−4**^	0.0083	0.9845		**<10**^**−4**^		0.2095
			**NASAL**	**<10**^**−4**^	0.2397			**<10**^**−4**^	0.0013	0.3536		**<10**^**−4**^		0.9127
			**VOICED**	**<10**^**−4**^	0.3769			**<10**^**−4**^	0.7524	0.1291		**<10**^**−4**^		0.0443
	Vow	Height	**OPEN**	0.0071	**<10**^**−4**^			**<10**^**−4**^	**<10**^**−4**^	0.8220		0.4099		0.1041
			**MID**	0.0045	0.0016			0.0085	**<10**^**−4**^	0.8384		0.6512		**<10**^**−4**^
			**CLOSE**	0.0237	**<10**^**−4**^			**<10**^**−4**^	**<10**^**−4**^	0.5913		0.3750		0.5593
			**ROUND**	0.8729	**<10**^**−4**^			0.1374	**<10**^**−4**^	0.5339		0.0074		**<10**^**−4**^
			**BACK**	0.0656	**<10**^**−4**^			**<10**^**−4**^	**<10**^**−4**^	0.4204		0.0187		**<10**^**−4**^

Movement type (hit, slide and ring), shape (rounded and spiky) and sound type (high and low frequency) were used as independent factors. A cutoff point p<0.00038 Bonferroni corrected is shown in boldtype.

Since phonological features describe vocal sounds in different spaces (acoustical, articulatory, anatomical, etc) many of them are not mutually exclusive. The matrix of correlations between them is shown in S2 Fig, and we refer to them when needed for interpretation purposes. The size variable was not included in the Table 1, as it did not reach significance for any phonological feature across modalities. The simplest explanation for this result is that the variable was not captured by our experimental design. We therefore discarded it for subsequent analyses.

**Fig 2:**
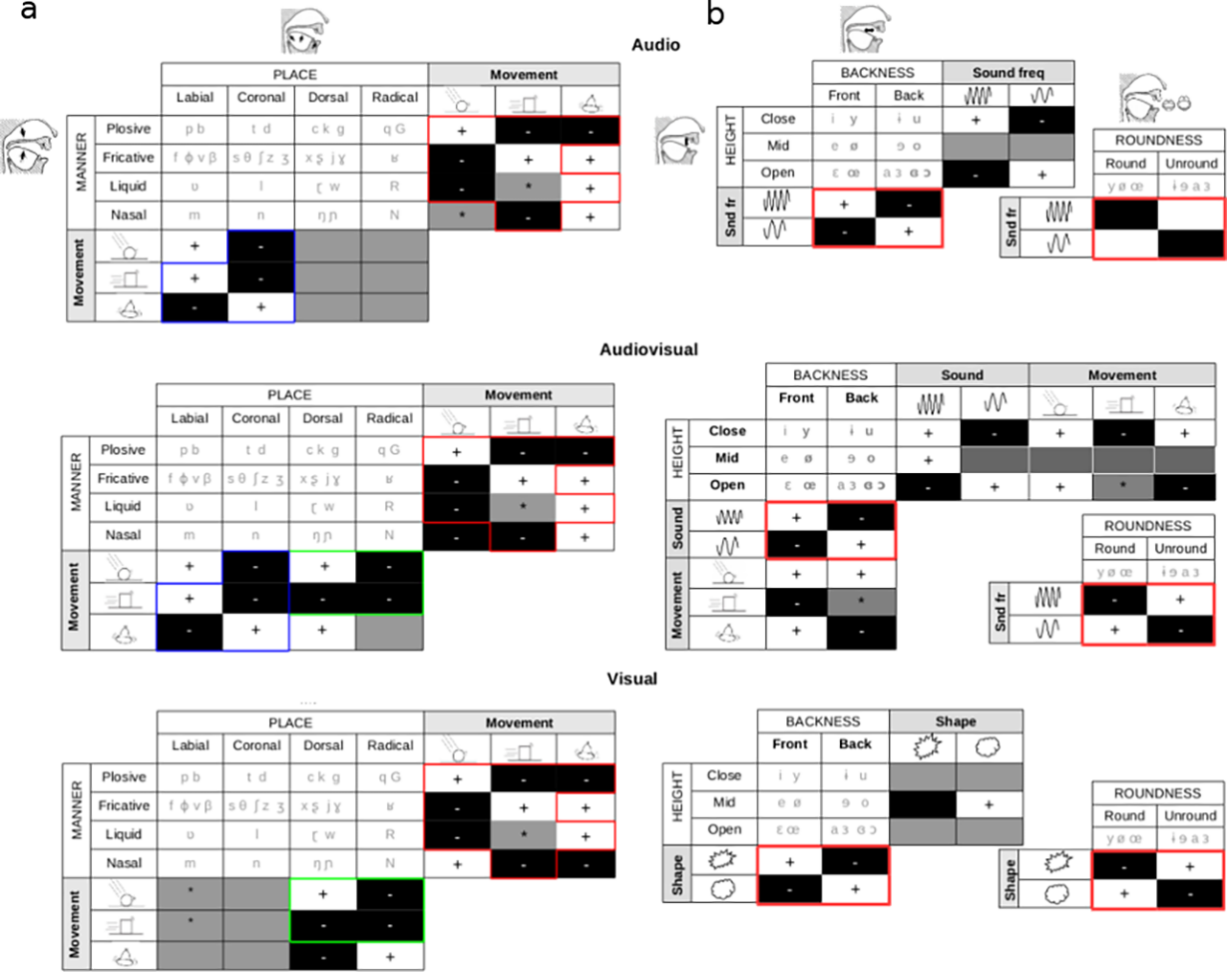
Movement types are communicated through consonants, shapes and sounds are communicated through vowels. a. White (+) and black (-) sites represent feature values greater than three and lesser than minus three s.d. from the modality mean, respectively. Gray is used for features that did not reach significance and also for feature values closer than three s.d. to the modality mean (marked with *). The phonological patterns for the different movements are highly conserved across sensory modalities through the manner of articulation of the consonants (red), making this phonological dimension a strong carrier for the type of movement: hits are characterized by plosives, slides by fricatives and rings by liquids and nasals. On the other hand, the patterns corresponding to the place of articulation vary across categories. Dorsals and radicals are used for V stimuli while labials and coronals for A stimuli. Both patterns act synergistically to characterize movements in AV (in green the pattern shared with V and in blue the one shared with A).**b.** White and black sites represent feature values greater than three and lesser than minus three standard deviations from the modality mean, respectively. The patterns corresponding to backness of the tongue and roundness of the lips are preserved through sensory modalities (red), representing shape in V and sound type in A and AV: spiky figures and high frequency sounds are dominated by unrounded and front vowels. Inversely, rounded figures and low frequency sounds are dominated by rounded and back vowels. In the multisensory AV case, the independence of phonological and perceptual dimensions is broken: movements have effects on both vowels and consonants.

Many perceptual dimensions present significant effects on distinctive features in the three modalities (upper part of Table 1). For movement type, the features that differ significantly in all three modalities are *nasal*, *consonantal* and *continuant* (these two last dimensions show a correlation of -0.2, p<0.001). The only one that is not included in IPA features is *consonantal*, precisely the one that separates vowels from consonants. Movement type has a significant effect on *consonantal* in every modality (marginal for A stimuli). For the AV case, hits and slides are represented by a low number of vowels, while rings are dominated by vowels (Fig 2). Indeed, the lower part of Table 1 shows a striking double dissociation between consonants and vowels. In the A and V modalities, movements have a significant effect on the selection of consonants and no effect on vowels; conversely, sounds and shapes have no effect on consonants, and significant effects on every vowel dimension.

The case is different for the multisensory modality AV, and associate two patterns. First, shapes have no effect on any consonant or vowel dimension. At this level of analysis, this aspect of the stimuli is lost in the novel onomatopoeias. Second, effects of sound and movements are present for both vowels and consonants, while those dimensions affected selectively one or the other class with unimodal stimuli.

The results of Table 1 can be summarized as follows: participants recruit differences in consonants to depict the structure of hit/slide/ring events. When sound frequency contrasts are involved as well, vowels are recruited for depicting that. If there is no sound but a rounded/spiky contrast is present, this frees up the vowel space for depicting that.

### Distinctive roles of consonants and vowels

To further characterize the phonological patterns elicited by the different movement types, we identified the IPA features which are away from the mean value by more than three standard deviations, separately for each sensory modality, as represented using color codes in Fig 2.

#### Consonants

Fig 2A shows that the manner of articulation is highly preserved across modalities (the preserved pattern is marked with red borders in Fig 2A): *hits* are characterized by plosives and a lack of fricatives and liquids; *slides* are dominated by fricatives, with very few plosives and nasals. *Rings* are dominated by liquids with a low proportion of plosives.

These analyses refine our previous observation that consonants are used to express movement type. Specifically, the manner of articulation is a robust carrier of movement information across A, V and AV modalities. The place of articulation, on the other hand, is more specific to each sensory channel: consonants articulated towards the back of the vocal tract were used for describing objects in motion (dorsals and radicals), and those articulated towards the front (labials and coronals) were used to characterize the sounds associated to the movements. Interestingly, the multisensory AV case presents both types of patterns, sharing visual and auditive information about the movements (in green and blue respectively in Fig 2B).

#### Vowels

ANOVAs revealed that movement has no effect on vowel selection for A and V stimuli, while it has a significant effect in all vocalic dimensions for AV stimuli. Shape has a significant effect on all the vowel dimensions for V and no effect for AV. Finally, sound has a significant effect on every dimension for A and AV.

Vowels present patterns that are highly preserved across sensory modalities (red in Fig 2B) for different shapes and sounds: low frequency sounds and rounded shapes are dominated by a majority of rounded and back vowels. The inverse corresponds to high frequency sounds and spiky shapes, represented by unrounded and front vowels. This agrees with the bouba-kiki effect for vowels, and unveils a cross-modal link between frequency and shape. The dimension of height is more variable across modalities, and its pattern is relatively preserved in A and AV modalities.

### Predicting stimulus features from onomatopoeias with machine learning

The ANOVA analyses allow finding associations between phonological features and perceptual aspects of the stimuli. However, these tests do not inform the sensitivity of the sensory-phonological correspondences at the single trial level or the possible synergistic effects of combining several features to improve this sensitivity. To answer these questions, we performed a classification-based analysis using a linear discrimination analysis (LDA). The algorithm learns a model to relate stimuli features to regions of the phonological space. This association is trained in a group of onomatopoeias (training set). Then, the performance of the model is quantified by comparing the real and model class prediction of onomatopoeias belonging to the complementary set (test set). The results are cross-validated, i.e. the process is repeated changing the test and trained sets until overall performance is fully quantified (see Methods for details). The overall performance is quantified using matrices that summarize for all onomatopoeias the actual and predicted classes and by quantifying the coefficient φ, which quantifies the prediction performance of the classifier. This algorithmic approach is complementary to ANOVA analyses and free of the biases that may appear in a listening and labeling task with humans.

**Fig 3:**
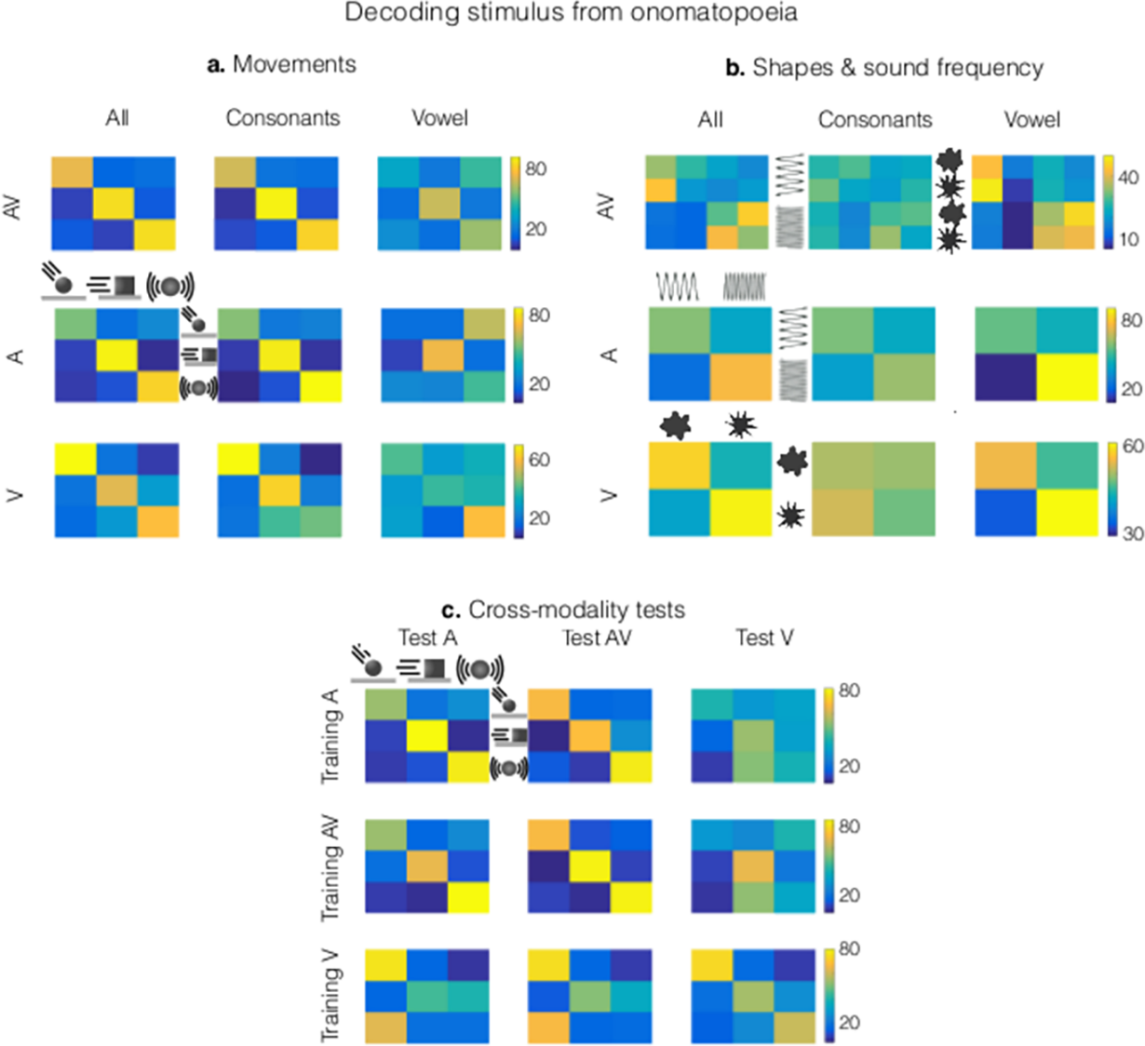
Decoding performance of the onomatopoeia within sensory modalities and their predictive power across sensory modalities. We used a machine learning algorithm to evaluate the performance of the novel onomatopoeias at classifying movements, shapes and sound frequencies. **a.** The system was trained to classify the movement types (hits, slides and rings). Each matrix contains the proportion of onomatopoeias classified in a given movement type (i.e. for all columns in the first row, hit onomatopoeias are respectively classified as hit, slide or ring). High and low decoding performances are yellow and blue respectively. The process is repeated for all modalities and using as features either all phonemes, only the consonants or only the vowels. Consonants produce better performances than vowels in all sensory modalities, with no synergistic effects for consonants and vowels taken together (except for the V condition). **b.** The system was trained to classify shapes and sound frequencies. Decoding performances are maximized using only the vowels, for all the sensory modalities. In the AV case, shape information is virtually lost (blue upper right and lower left blocks). Decoding performances of rounded shapes producing low frequency sounds and spiky shapes producing high-frequency sounds are enhanced, replicating the phonological link between shapes and sound frequencies already found using ANOVA (Fig 2B, A and V panels). **c.** Cross-modality tests. The system was trained with the onomatopoeias of one sensory-modality and tested in each other modality. S1 Table contains the numerical values of the decoding accuracies.

First, we used LDA to both train and test the model within the same modality (A, V or AV). The results obtained with LDA are consistent with the ones obtained with the ANOVAs, as summarized in the matrices of Fig 3.

Fig 3A shows the decoding performance for movement type (hits, slides and rings). When only consonants are used as features to train the LDA, decoding performances (φ = 0.68±0.04) are higher than the ones obtained when using only vowels (φ = 0.25±0.03). No synergistic effects are present for vowels and consonants taken together (φ = 0.68±0.06), except for the V condition (slides and rings are better classified using vowels and consonants). Fig 3B shows the decoding performances for shapes and sound frequencies. The role of vowels and consonants is inverted with respect to the previous case: performances are better for vowels (φ = 0.48±0.02) than for consonants (φ = 0.20±0.08), with no synergistic effects for vowels and consonants taken together (φ = 0.36±0.06). Shapes are almost indistinguishable in the AV condition, while the mean sound frequencies are relatively well decoded (see S1 Table for values).

We then used the classifier to test the predictive power of movement type using onomatopoeias across sensory modalities. We explored whether an onomatopoeia associated with a given movement in one sensory modality is also representative for that movement when perceived in another modality. For instance, we can test if the onomatopoeias produced for the video of a sliding object with no sound is also good for describing a sound of a sliding object. To address this, we trained the classifier in one modality and tested it in every other one. The results (Fig 3C) show that onomatopoeias of the different movement types produced in A modality are good representations for the movements in AV (φ = 0.56±0.06) and poor representations for movements in V (φ = 0.12±0.2). Furthermore, onomatopoeias for AV movements are good representations for A movements (φ = 0.57±0.1) and poor representations for V movements (φ = 0.25±0.2). Onomatopoeias produced for V movements do not generalize to the ones produced for the other modalities.

It has been shown that individual differences may be important for cross-modal processing in sound symbolism [24,25]. To check for individual differences, we set up an LDA using a single onomatopoeia per speaker, averaging all his/her onomatopoeias across each sensory modality. We obtained the same general results as the ones presented here (i.e. movements are better decoded by consonants and shapes and frequencies by vowels, with no synergistic effects), building confidence in the robustness of our results across participants.

### Application to cross-linguistic onomatopoeias

The analyses presented so far concern novel onomatopoeias produced extemporaneously in a controlled experiment. Here we used the model trained with our onomatopoeias as a movement-type classifier for a list of cross-linguistic onomatopoeias.

The list was extracted from Wikipedia [26] and restricted to actions performed by objects (human and animal actions were excluded) for which onomatopoeias in at least 10 languages were reported (S5 File). A note of caution is needed here. First, since no phonetic convention was followed for the words listed on the Wikipedia article, the orthographic forms were treated as if they were IPA transcriptions. Second, given that many languages are historically related, further control for the Galton problem is needed. Although these problems impose serious constraints to interpreting the results of this application in its present version, this kind of method might be useful for future comparative investigations of cross-linguistic data.

**Fig 4:**
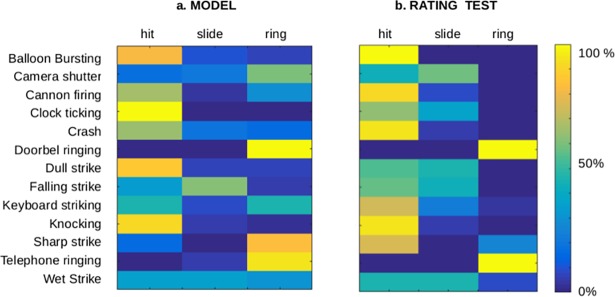
Decoding the movement type of cross-linguistic onomatopoeias. **a.** We train the LDA model with the AV onomatopoeias and used it as a movement-type classifier (hit, slide or ring) for the cross-linguistic onomatopoeias extracted from Wikipedia [26]. The list was restricted to non-human/animal actions with onomatopoeias in 10 languages for each action (balloon bursting, camera shutter, etc). The color code corresponds to the percentage of languages in which onomatopoeias are classified in each movement category. b. The same actions were classified by a group of 20 human raters, showing good agreement with the model-derived predictions.

Fig 4A shows that many actions are classified as single movements: crashes, knocking and dull strikes are classified as hits, and telephone rings and doorbells as rings. Beyond these cases, complex actions are more distributed across languages. For instance, falling strikes are classified as hits (as teok in Korean) or slides (as pljas in Croatian), reflecting both movement components (a strike and a fall). Also, the complex action of a wet strike is characterized by a uniform distribution of hits (as bicha in Japanese), slides (as splash in English) and rings (as plons in Afrikaans). In Fig 4B we show the same actions classified by a group of human raters (see Methods). They were asked to give the proportions of a hit, a slide and a ring they think were present in each action. The results of the model-derived predictions are in good agreement with the human ratings (*r* = 0.72, *p*<0.001), including the distribution of complex actions across movements, as in the cases of falling and wet strikes.

## Discussion

In this work we analyzed the phonological structure of novel onomatopoeias produced to describe audio, visual and audiovisual moving and interacting objects. We used images of different shapes performing different movements, accompanied with sounds centered either on a low or a high frequency.

Dingemanse and collaborators noted that in the investigation of form-meaning mappings through behavioral experiments, “studies have relied on forced-choice methods with non-word pairs constructed for maximal contrasts, which provides a reason for caution in interpreting the results” [3]. Also, these studies often reduced the investigation to a single perceptual dimension, as the shape of an object or its movement. Here we overcome these limitations by letting the participants create novel onomatopoeias of different interacting objects without any constraints.

The speech recordings were then transcribed to IPA alphabet and quantified by a vector of basic phonological features. Using independent data analysis techniques including ANOVAs and machine learning algorithms, we found that different perceptual aspects of the moving objects are communicated through specific phonological dimensions of onomatopoeias.

A main finding is that movements are coded through consonants. This fits well with previous theoretical attempts to relate physical events involving rigid objects (hits, slides and rings) with broad groups of speech sounds (plosives, fricatives and voiced, respectively) [21]. We found that the manner of articulation in the vocal tract (occluded, constricted, etc) is a robust dimension encoding movement types for A, V and AV stimuli: hits are dominated by plosives, with a low proportion of fricatives and liquids; slides are dominated by fricatives and a low proportion of plosives and nasals; finally, rings are dominated by liquids and a lower proportion of plosives.

The finding that consonants code movement types imposes a rather strong constraint to the transmission of the other perceptual aspects of the stimuli. For instance, it is known that participants are sensitive to both vowel and consonant content when labeling objects of different shapes, using sonorant consonants and rounded vowels for rounded images and plosive consonants and unrounded vowels for spiky ones [16]. Moreover, consonants have been found to be more important than vowels in the bouba-kiki effect [15]. While this holds for static objects, we show here that vowels are the principal carriers for depicting shapes of moving objects through onomatopoeias. Although much care is needed to generalize results of sound symbolism based on controlled experiments to natural languages, it is tempting to compare our perspective with the analysis performed on nearly two-thirds of the world’s languages in [2]. They showed that basic vocabulary items carry strong associations with specific kinds of speech sounds, for instance *round* often appears with an *r* and *small* with an *i*. It would be interesting to explore to what extent these associations are conserved and others appear in a context of complex sensory stimuli.

The sets of vowels used by our participants are compatible with previous results reporting that rounded vowels are used for smooth objects and unrounded vowels for spiky ones. Here we found that smooth and spiky objects are also characterized by back and front vowels respectively.

Interestingly, the phonological patterns for round vs spiky shapes in V stimuli are also used to code mean sound frequencies in A stimuli: rounded and back vowels code low frequency sounds, while high frequency sounds are coded by unrounded and front vowels. Vowels are characterized by the first two vocal tract resonances or formants, F1 and F2 [27]. Front vowels have higher second resonant frequencies F2 than back vowels, which offers a possible explanation of the use of front and back vowels for coding sound frequency from a strictly acoustical point of view.

The relationship between the multisensory AV and the single-sensory A and V cases is not trivial. First, the shapes are no longer coded for AV stimuli. Second, the general observation that consonants and vowels code different perceptual dimensions in A and V stimuli does not hold anymore with AV stimuli. In unisensory conditions the consonants are used to describe the type of movement, and the vowels to describe shapes in V and sound frequencies in A. In contrast, in the multisensory AV case, movements are coded by both vowels and consonants. This is shown in Figs 2B and 3B, where consonants alone and vowels alone code the different movement types, while vowels are weak carriers of movement type for A and V stimuli.

One of the limitations of this study is that the temporal structure of the produced onomatopoeias was left out of the analysis. The averaging procedure used to compare onomatopoeias in the phonological space precludes from analyzing the iconic effects of operations widely used in creative depiction, as reduplication and lengthening of segments [7]. A natural follow-up of this work would be to explore what is encoded by the temporal structure of the novel onomatopoeias and if this code is general across different sensory modalities and languages.

The structure of onomatopoeias and, more generally, the influence of vocal imitation in word formation has historically received poor attention [28]. Only recently, evidence has accumulated towards the idea that the onomatopoeia is one pertinent object to study the evolution of language, integrating the physics of the vocal system [4,29] and the neural basis of multisensory brain processing [30,31]. We believe that an exploration of the onomatopoeic structure merging mathematical modeling of the vocal system, perceptual experiments and neural processing techniques will help building a new program for the study of language from a biophysical standpoint.

## Materials and methods

### Participants

A total of 19 native French speakers (10 females, aged 20–35, mean 27.5 years) completed the experiment. All subjects were free of communication disorders and passed audiometric screening.

Another 20 participants (12 females, aged 22–45, 10 English speakers, 10 Spanish Speakers) completed a rating test. Specifically, they were asked to "write down the percentage of a hit, a slide and a ring that you think are present in each of the following actions", for the actions listed in Fig 4.

All subjects were paid for their participation in the study and signed an informed consent. The experiments were approved by the Ethical Committee of the Kremlin-Bicêtre hospital APHP (no. 98–25).

### Stimuli

The stimuli were constructed using two basic objects (rounded and spiky) of two sizes (big and small) and three basic movements (*slide* of one object on a horizontal surface, *hit* between objects coming from opposite directions and a *ringing* object). The stimuli were generated and presented using *Psychtoolbox* under MATLAB [32], (S1 File).

The experiment was divided in three blocks of different sensory modalities:

Visual (V). We generated 12 videos = 2 shapes × 2 sizes × 3 movements. Videos were 4s length.

Audio (A). Sound files corresponding to a slide, a hit and a ring were downloaded from www.audiomicro.com. The audio files were manipulated using Praat [33] to obtain a low-frequency (LF) and a high-frequency (HF) version of each sound. To do so, the spectral center of each sound was moved to 1.1 kHz for LF and 3.0 kHz for HF using the Praat function *Modify-override*. The original sound length was recovered using the function *Convert-Lenghten*. This procedure yielded a total of 6 audio files = 3 movements × 2 sounds.

Audiovisual (AV). Stimuli consisted in every combination of visual and auditory stimuli (sounds were congruent with movement types). This design yielded a total of 24 videos = 2 shapes × 2 sizes × 3 movements × 2 sounds.

The final database consisted of 798 recorded onomatopoeias = 19 subjects × (6A + 12V + 24AV).

Participants sat in a silent room, 0.5m away from a 23-inch led monitor. Visual stimuli were presented in black over a white background. The angle subtended by the big and small figures was 6° and 1° respectively. The sounds were sampled at 22.05 kHz, were set to the same overall energy (RMS) and presented through headphones. The complete set of stimuli within each block was randomized before presented to the participants.

### Task

The presentation order of the A, V and AV blocks was randomized, as well as the stimuli within each block. After the presentation of each stimulus, participants were asked to pronounce the onomatopoeic non-word that would better represent the stimulus. They were allowed to repeat the presentation of the stimuli as they wished by pressing a key.

### Statistical analyses

The audio files were processed as follows:

#### Transcription

The speech files generated by the participants were phonetically transcribed to IPA. Some onomatopoeias were discarded due to inter-transcriber differences or low sound intensity. The final table of 791 transcribed onomatopoeias can be found in S2 File and S3 File.

#### Projection to distinctive features

Each phoneme was decomposed into the binary space of distinctive features, as described in [23] (S4 File and S6 File). Each onomatopoeia was therefore mapped to a binary matrix whose rows correspond to the phonemes and columns to phonetic features. The matrix was then collapsed to a 12-dimensional array by averaging across its rows (see Fig 1).

#### Machine learning

Each onomatopoeia was characterized by its phonemes projected into the 13 IPA features: radical, dorsal, coronal and labial (consonantal places), plosive, fricative, liquid and nasal (consonantal manners), open, mid and close (vowel height), round and back. We then used machine learning with a linear discriminant analysis LDA (coded in MATLAB available in S7 File) as a classifier to calculate the performance of the onomatopoeias at predicting the movement types (hit, slide, ring), shapes (rounded and spiky) and frequency (high and low). A 10-fold cross-validation, repeated 1000 times, was used to estimate the performance of the classifier. Ultimately, this procedure generated 1000 repetitions of a matrix containing the percentage of times a given class (i.e. hit) was predicted to a given class (i.e. slide). These matrices where reduced to only one by computing the median and inter-quartile-range (IQR) across repetitions (S1 Table). This procedure was repeated using either all features (*n* = 13), only the consonant features (*n* = 8) or only the vowels features (*n* = 5) and it was also repeated for the three types of modalities (A, V and AV).

A similar procedure was followed for cross-modality predictions. In this case, the classifier was trained in a given modality (i.e. audio) and tested in the other modalities (visual and audio-visual) using vowels and consonants as features for the classifier. In order to make the results of the cross-modality comparable to those of the within-modality, an identical procedure was followed in both cases. Ten different classifiers where estimated for each given class using the cross-validation 10-folds from the within-class analysis. These classifiers where then used to predict the classes of the onomatopoeias produced in the other modalities. Again, the whole procedure was repeated 1000 times. Ultimately, this yields for each analysis where the training occurred in the X modality and the testing in the Y modality, a total of 10×1000 matrices. These matrices where reduced to only one by computing the mean in the first dimension (10) and the median and IQR on the second dimension (1000).

We made sure that the onomatopoeias produced by one speaker where either on the training set or the test set. By doing so we avoided overfitting our results by using some of the onomatopoeias of one speaker to predict the rest of his/her production.

The classification performance of each individual matrix was quantified using the Matthews correlation coefficient, known as φ [34]. This metric takes into account the true and false identification of the different classes predicted by the classifier. The φ coefficient ranges from +1 to -1, where +1 represents perfect classification, 0 equal to random classification and -1 complete disagreement between prediction and true labels. Multiclass classifications (such as the three movements evaluated in Fig 3A) where quantified using a “Macro-averaging” strategy. For each class the performance was individually quantified (i.e. A versus not A, B versus not B, etc) and the overall φ coefficient was computed from the average of the individual coefficients.

We used a cross-linguistic list [26] containing onomatopoeias characterizing different objects in action (excluding human and animal actions), for which onomatopoeias were reported in 10 languages at least (S8 File). The words in the list were directly translated to IPA. Onomatopoeias were predicted as matching one of the studied movements (slide, hit or ring) using the classifier originally derived from audiovisual modality that that was trained using as features all the phonemes.

## Supporting information

S1 File Audiovisual stimuli used in our experiments. (RAR)

S2 File List of the onomatopoeias produced by each participant, sensory modality and stimulus type transcribed to IPA. (RAR)

S3 File Reference code used in S2 File for each stimulus type. (RAR)

S4 File 12-phonological feature decomposition of each IPA symbol. (RAR)

S5 File Proportions of hit, slide and ring present in the actions listed in the Wikipedia, rated by a population of 20 participants. (RAR)

S6 File Matlab codes that map the IPA-onomatopoeias into the phonological space and performs the statistical tests. (RAR)

S7 File Matlab implementation of machine learning with a linear discriminant analysis LDA as a classifier to predict the movement types (hit, slide, ring), shapes (rounded and spiky) and frequency (high and low) from their onomatopoeia. (RAR)

S8 File Matlab code for computing the correlation between the LDA model and the human ratings for the actions listed on the cross-linguistic onomatopoeias. (RAR)

S1 Fig Phonemes are broken down to basic phonological features (International Phonetic Alphabet and Distinctive Features). The audio files of the onomatopoeias created by the participants were transcribed to the symbols of the International Phonetic Alphabet. The complete set of phonemes used by the participants is shown in the 2-dimensional charts for consonants (upper panel) and vowels (lower panel). There are a few sites for which there are two phonemes. In those cases, both phonemes were absorbed into one single phoneme (the first of the pair). This was done to univocally associate each phoneme to a point in the feature space without increasing the number of phonological dimensions needed to discriminate them. In the IPA space (dark grey), consonants are characterized by their *place of articulation* in the vocal tract and their *manner of articulation*. Vowels are defined by the *heightness* and *backness* of the tongue. The distinctive features (light gray) are the lowest phonological features from which phonemes can be built of, and can be combined to generate the IPA features.
(TIF)

S2 Fig Correlation matrix for phonological features. Phonological features describe properties of the vocal sounds in acoustical, articulatory and anatomical dimensions, which are not mutually exclusive. For instance, the sounds produced using the nasal tract (*nasal*) use the vocal folds as a sound source (*voiced*). These features are therefore posivitely correlated.
(TIF)

S1 Table Decoding performances of the A, V and AV stimuli. Decoding accuracies (median±iqr) for the matrices shown in Fig 3. The corresponding φ coefficient can be found under each table.
(DOCX)
