# Supplementary figures and images for: The audiovisual structure of onomatopoeias: An intrusion of real-world physics in lexical creation
Source: PLoS One. 2018 Mar 21;13(3):e0193466. doi: 10.1371/journal.pone.0193466 (PMC5862436; doi:10.1371/journal.pone.0193466)

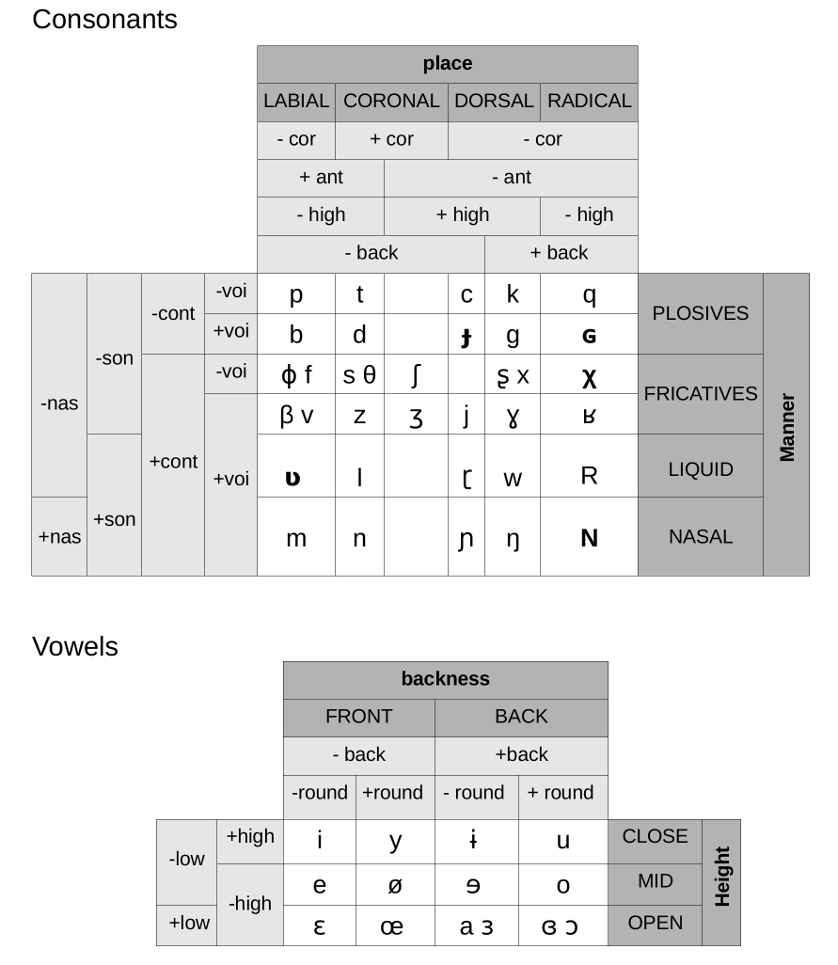

Supplement: S1 Fig — The audio files of the onomatopoeias created by the participants were transcribed to the symbols of the International Phonetic Alphabet. The complete set of phonemes used by the participants is shown in the 2-dimensional charts for consonants (upper panel) and vowels (lower panel). There are a few sites for which there are two phonemes. In those cases, both phonemes were absorbed into one single phoneme (the first of the pair). This was done to univocally associate each phoneme to a point in the feature space without increasing the number of phonological dimensions needed to discriminate them. In the IPA space (dark grey), consonants are characterized by their place of articulation in the vocal tract and their manner of articulation. Vowels are defined by the heightness and backness of the tongue. The distinctive features (light gray) are the lowest phonological features from which phonemes can be built of, and can be combined to generate the IPA features. (TIF) [file pone.0193466.s009.tif]

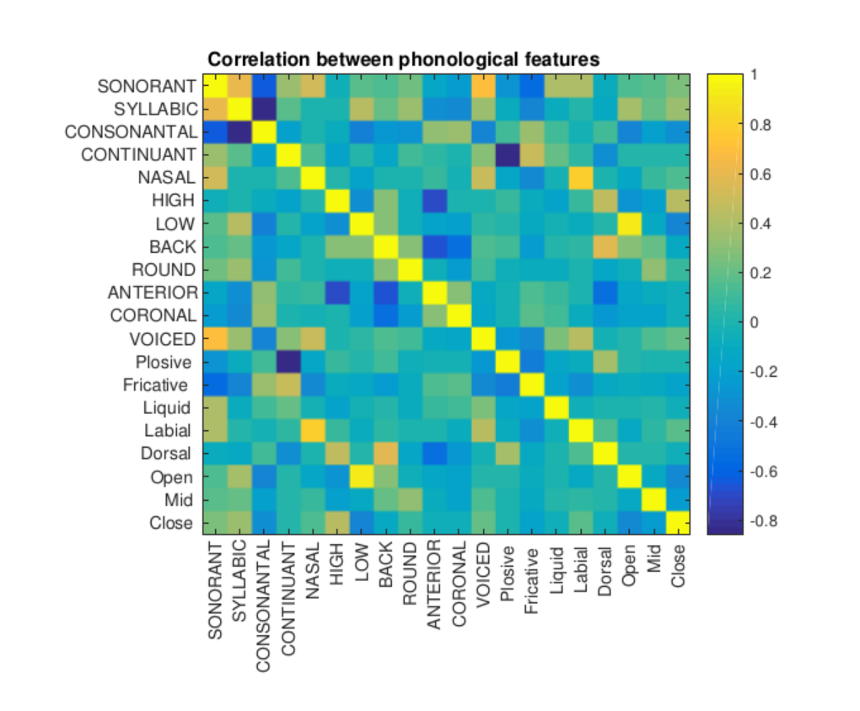

Supplement: S2 Fig — Phonological features describe properties of the vocal sounds in acoustical, articulatory and anatomical dimensions, which are not mutually exclusive. For instance, the sounds produced using the nasal tract (nasal) use the vocal folds as a sound source (voiced). These features are therefore posivitely correlated. (TIF) [file pone.0193466.s010.tif]
