# Supplementary material for: The audiovisual structure of onomatopoeias: An intrusion of real-world physics in lexical creation
Source: PLoS One. 2018 Mar 21;13(3):e0193466. doi: 10.1371/journal.pone.0193466 (PMC5862436; doi:10.1371/journal.pone.0193466)
Supplement: S1 Table — Decoding accuracies (median±iqr) for the matrices shown in Fig 3. The corresponding φ coefficient can be found under each table. (DOCX) [file pone.0193466.s011.docx]

**a. All Consonants Vowels**

|  |  | **Hit** | **Slide** | **Ring** |  |  |  |  |  |  |  |  |
| --- | --- | --- | --- | --- | --- | --- | --- | --- | --- | --- | --- | --- |
|  | **Hit** | 70.9±1.3 | 13.2±1.3 | 15.9±0.7 |  | 67.5±1.3 | 17.2±1.3 | 15.2±0.7 |  | 34.4±2.0 | 19.9±1.3 | 46.4±2.0 |
| **AV** | **Slide** | 5.9±0.7 | 82.9±1.3 | 11.2±1.3 |  | 3.3±1.3 | 87.5±1.3 | 9.2±0.7 |  | 16.4±2.0 | 65.1±1.3 | 18.4±2.0 |
|  | **Ring** | 10.2±0.7 | 6.8±0.7 | 83.0±0.7 |  | 8.1±0.7 | 10.2±1.4 | 81.6±1.3 |  | 25.2±2.0 | 16.3±0.7 | 58.5±2.0 |
|  |  |  | **φ=0.68±0.06** |  |  |  | **φ=0.68±0.05** |  |  |  | **φ=0.25±0.3** |  |
|  |  | 52.8±5.5 | 19.4±2.8 | 27.8±2.8 |  | 55.6±2.8 | 22.2±2.8 | 25.0±2.8 |  | 19.4±5.5 | 19.4±.8 | 61.1±5.5 |
| **A** |  | 11.8±2.9 | 82.3±5.9 | 5.6±2.9 |  | 8.8±2.9 | 82.3±5.9 | 8.8±2.9 |  | 11.8±5.9 | 70.6±2.9 | 20.6±2.9 |
|  |  | 8.6±2.9 | 14.3±2.9 | 77.1±5.7 |  | 2.9±2.9 | 11.4±2.9 | 82.9±2.9 |  | 25.7±8.6 | 25.7±2.9 | 48.6±8.6 |
|  |  |  | **φ=0.58±0.1** |  |  |  | **φ=0.61±0.1** |  |  |  | **φ=0.09±0.4** |  |
|  |  | 73.6±2.8 | 18.0±2.8 | 8.3±1.4 |  | 73.6±1.4 | 20.8±1.4 | 5.6±0.0 |  | 40.3±2.8 | 26.4±4.1 | 33.3±2.8 |
| **V** |  | 17.8±2.7 | 54.8±4.1 | 27.4±4.1 |  | 16.4±2.7 | 64.4±4.1 | 19.2±4.1 |  | 27.4±1.3 | 38.4±0.0 | 34.2±1.4 |
|  |  | 15.7±1.4 | 25.7±4.3 | 58.6±4.3 |  | 17.1±1.4 | 40.0±1.4 | 42.9±2.9 |  | 27.1±2.9 | 12.9±2.9 | 60.0±2.9 |
|  |  |  | **φ=0.42±0.2** |  |  |  | **φ=0.36±0.2** |  |  |  | **φ=0.2±0.1** |  |

**b. All Consonants Vowels**

|  |  | | **Low Freq** | | **High Freq** | |  |  |  |  |  |  |  |  |  |  |
| --- | --- | --- | --- | --- | --- | --- | --- | --- | --- | --- | --- | --- | --- | --- | --- | --- |
|  |  | | **Round** | **Spiky** | **Round** | **Spiky** |  |  |  |  |  |  |  |  |  |  |
| **AV** | **LF** | **R** | 33.6±2.6 | 27.4±3.5 | 21.7±2.6 | 16.8±2.6 |  | 26.5±3.5 | 29.2±3.5 | 21.2±3.5 | 23.0±3.5 |  | 43.3±5.3 | 15.0±5.3 | 24.8±1.8 | 16.8±1.8 |
|  |  | **S** | 42.9±4.4 | 19.6±4.0 | 16.9±3.6 | 20.5±2.7 |  | 31.2±4.5 | 22.3±3.6 | 19.6±3.6 | 26.8±3.6 |  | 49.1±2.7 | 7.1±1.8 | 25.0±2.7 | 18.7±1.8 |
|  | **HF** | **R** | 14.0±2.6 | 11.4±2.6 | 29.8±3.5 | 44.7±3.5 |  | 25.4±3.5 | 16.7±3.5 | 28.1±3.5 | 29.8±3.5 |  | 15.8±1.7 | 3.5±2.6 | 35.1±3.5 | 45.6±3.5 |
|  |  | **S** | 12.6±2.7 | 11.7±2.7 | 42.3±3.6 | 33.3±3.6 |  | 27.0±2.7 | 17.1±3.6 | 33.3±4.5 | 22.5±4.5 |  | 15.3±1.8 | 4.5±2.2 | 38.7±3.6 | 41.4±3.6 |
|  |  |  | **φ=0.05±0.02** | | | |  | **φ= -0.006±0.02** | | | |  | **φ=0.08±0.02** | | | |

|  |  | **Low Freq** | **High Freq** |  |  |  |  |  |  |
| --- | --- | --- | --- | --- | --- | --- | --- | --- | --- |
| **A** | **Low Freq** | 59.6±3.8 | 40.3±3.8 |  | 57.7±3.8 | 42.3±3.8 |  | 55.8±1.9 | 44.2±1.9 |
|  | **High Freq** | 24.5±1.9 | 75.5±1.9 |  | 37.7±3.8 | 62.3±3.8 |  | 11.3±1.9 | 88.7±1.9 |
|  |  | **φ=0.36±0.06** | |  | **φ=0.2±0.08** | |  | **φ=0.48±0.02** | |

|  |  | **Spiky** | **Rounded** |  |  |  |  |  |  |
| --- | --- | --- | --- | --- | --- | --- | --- | --- | --- |
| **V** | **Spiky** | 56.9±3.7 | 43.1±3.7 |  | 50.5±3.7 | 49.5±3.7 |  | 54.1±1.8 | 45.9±1.8 |
|  | **Rounded** | 40.6±3.8 | 59.4±3.8 |  | 52.8±4.7 | 47.2±4.7 |  | 33.9±5.7 | 66.0±5.7 |
|  |  | **φ= 0.16±0.05** | |  | **φ= -0.02±0.06** | |  | **φ= 0.21±0.05** | |

**c. A AV V**

|  |  | **Hit** | **Slide** | **Ring** |  |  |  |  |  |  |  |  |
| --- | --- | --- | --- | --- | --- | --- | --- | --- | --- | --- | --- | --- |
|  | **Hit** | 52.8±5.5 | 19.4±2.8 | 27.8±2.8 |  | 66.5±0.6 | 16.2±0.4 | 17.3±0.5 |  | 39.2±0.8 | 28.9±0.7 | 32.1±0.7 |
| **A** | **Slide** | 11.8±2.9 | 82.3±5.9 | 5.6±2.9 |  | 5.0±0.5 | 68.0±0.5 | 26.9±0.4 |  | 15.8±0.7 | 53.1±0.8 | 31.1±0.5 |
|  | **Ring** | 8.6±2.9 | 14.3±2.9 | 77.1±5.7 |  | 13.3±0.7 | 9.5±0.6 | 77.3±0.5 |  | 9.6±0.7 | 51.7±0.7 | 38.7±0.7 |
|  |  |  | **φ=0.58±0.1** |  |  |  | **φ=0.56±0.06** |  |  |  | **φ=0.12±0.2** |  |
|  |  | 56.7±0.6 | 16.4±0.3 | 26.7±0.3 |  | 70.9±1.3 | 13.2±1.3 | 15.9±0.7 |  | 30.4±0.4 | 27.2±0.3 | 42.4±0.4 |
| **AV** |  | 19.4±0.6 | 67.6±0.3 | 12.9±0.3 |  | 5.9±0.7 | 82.9±1.3 | 11.2±1.3 |  | 10.8±0.5 | 67.7±0.7 | 21.5±0.3 |
|  |  | 9.4±0.6 | 6.9±0.3 | 83.7±0.9 |  | 10.2±0.7 | 6.8±0.7 | 83.0±0.7 |  | 8.6±0.0 | 55.4±0.1 | 36.0±0.1 |
|  |  |  | **φ=0.57±0.1** |  |  |  | **φ=0.68±0.06** |  |  |  | **φ=0.25±0.2** |  |
|  |  | 75.8±0.8 | 16.4±0.8 | 7.8±0.6 |  | 74.4±0.5 | 16.2±0.5 | 9.4±0.3 |  | 73.6±2.8 | 18.0±2.8 | 8.3±1.4 |
| **V** |  | 15.9±0.9 | 44.1±1.5 | 40.0±1.2 |  | 14.1±0.7 | 50.8±0.9 | 35.1±0.7 |  | 17.8±2.7 | 54.8±4.1 | 27.4±4.1 |
|  |  | 62.6±0.9 | 18.9±0.6 | 18.6±0.9 |  | 66.1±0.8 | 16.5±0.4 | 17.4±0.8 |  | 15.7±1.4 | 25.7±4.3 | 58.6±4.3 |
|  |  |  | **φ=0.28±0.4** |  |  |  | **φ=0.33±0.4** |  |  |  | **φ=0.42±0.2** |  |
